# International diet quality index and revised diet quality index relationship with non-alcoholic fatty liver disease: a case-control study

**DOI:** 10.1186/s12876-023-03082-x

**Published:** 2023-12-14

**Authors:** Narjes Najibi, Mitra Kazemi Jahromi, Farshad Teymoori, Hossein Farhadnejad, Ammar Salehi-Sahlabadi, Parvin Mirmiran

**Affiliations:** 1 Department of Nutrition, School of Public Health, Iran University of Medical Sciences, Tehran, Iran; 2 Endocrinology and Metabolism Research Center, Hormozgan University of Medical Sciences, Bandar Abbas, Iran; 3 Nutrition and Endocrine Research Center, Research Institute for Endocrine Sciences, Shahid Beheshti University of Medical Sciences, Tehran, Iran; 4 Department of Clinical Nutrition and Dietetics, Faculty of Nutrition and Food Technology, National Nutrition and Food Technology, Shahid Beheshti University of Medical Sciences, Tehran, Iran

**Keywords:** Dietary pattern, Diet quality, Non-alcoholic fatty Liver Disease, Adults

## Abstract

**Background/objectives:**

Recent studies show the potential role of dietary quality and quantity in predicting the risk of chronic diseases, such as liver disease, therefore, in the present study, we aimed to assess the association of diet quality index International (DQI-I) and diet quality index revised (DQI-R) and their main components with the odds of non-alcoholic fatty liver disease (NAFLD) in adult populations.

**Methods:**

The current case-control study was conducted on 225 patients with NAFLD and 450 controls. Dietary intake was assessed using a validated 168-item food frequency questionnaire, and DQI-I and DQI-R were calculated in participants. Multi-variable logistic regression was used to examine the association of DQI-I and DQI-R and their components with the odds of NAFLD.

**Results:**

The mean (SD) age and BMI of participants (53% men) were 38.1 (8.8) years and 26.8 (4.3) kg/m^2^, respectively. In the final model, each one SD increment in the DQI-I score was associated with decreased odds of NAFLD (OR = 0.63; 95%CI = 0.40–0.98) (*P* = 0.040). Also, of DQI-I components, high adequacy score was related to lower odds of NAFLD (OR = 0.16; 95%CI = 0.06–0.40) (*P* < 0.001). Also, our findings showed that participants in the highest tertile of DQI-R score had lower odds of NAFLD compared to the lowest tertile of DQI-R (OR = 0.24; 95%CI = 0.10–0.53) (*P* < 0.001). Furthermore, of DQI-R components, high moderation score are linked to a lower risk of NAFLD (OR = 0.17;95%CI = 0.07–0.43) (*P* < 0.001).

**Conclusion:**

Our findings suggested that greater adherence to diet with a higher score of DQI-I and DQI-R may be associated with lower odds of NAFLD.

## Introduction

Non-alcoholic fatty liver disease (NAFLD) prevalence has been increasing worldwide. A recent study using 245 articles involving 2,699,627 persons forecast that NAFLD prevalence will be 55.7% by 2040, a three-fold increase since 1990 and a 43.2% increase from 2020 [1]. Globally, the NAFLD-related deaths and disability-adjusted life years (DALYs) in 2019 increased by 80.2% and 62.9% compared with 1990, respectively [2]. Besides obesity, genetics, and inactivity, dietary factors are recognized as the main risk factors for NAFLD [3, 4]. Different aspects of diet such as nutrients [5], food items [6], food groups [7, 8], and dietary indices [9, 10] were shown to be associated with NAFLD. One important aspect of a diet is diet quality which is defined in different types and many pre-defined indices developed to measure the quality of a diet.

The diet quality index(DQI) was introduced by Patterson et al. in 1994 based on dietary recommendations from the 1989 National Academy of Sciences publication Diet and Health [11]; they created DQI using the composition of dietary intake of 8 components (total fat, saturated fat, cholesterol, fruit and vegetables, grains and legumes, protein, sodium, and calcium). Five years later, Haines et al. developed a revised version of DQI as DQR-R [12] based on dietary assessment using two completed 24-hour recalls in 3202 adults. DQI-R is composed of 10 components including 4 of the original DQI components (total fat, saturated fat, cholesterol, and calcium), fruit and vegetable as separate components, grains as a single category, iron that was replaced with protein, and dietary moderation and diversity as new components. Also, the reproducibility and validity of the DQI-R which was assessed by the food frequency questionnaire (FFQ) instrument confirmed later [13].

In 2003, Kim et al. developed a new version of DQI as DQI International (DQI-I) using the dietary data of the Chinese and US populations for global monitoring and exploration of diet quality across countries [14]. The DQI-I was developed based on four main categories including a variety of food groups, adequacy of some nutrients and food groups, moderation in intake of some nutrients, and overall balance in macronutrients and different types of fatty acids(FAs).

Over the past two decades, different research was designed to introduce and validates the DQI for different population groups based on periods of life such as children, adolescent, and elderly [15, 16], pregnant [17], or different diseases such as colorectal cancer [18] and diabetes(DM) [19], or different region such as DQI for Brazilians [20]. However, DQI as an instrument of diet quality, in a smaller number of studies has been assessed with the risk of cardiometabolic factors [21–28]. Funtikova et al. during a 10-year follow-up in adults indicated that higher DQI was beneficially related to lipid profile and waist circumference (WC) [21]. In another study in adolescents, higher adherence to a DQI adapted for Brazilian adolescents was associated with a better cardiometabolic profile in girls with normal weight; not in overweight/obese girls. In boys with overweight/obesity, higher DQI was associated with lower concentrations of total cholesterol and low-density lipoprotein cholesterol (LDL-c), but only LDL-C remained significant in boys with normal weight [24]. However, in a case-control study, Brazilian DQI revised and its components have not been associated with the development of breast cancer risk [22].

Studies investigating the DQI-I relationship with cardiometabolic factors are scarce. Setayeshgar and her colleagues indicated that a 10-unit improvement in DQI-I score was associated with lower gain in central fat mass and body fat percent after a 2-year follow-up in Canadian children [25]. In another study, DQI-I was not associated with BMI and obesity in Chinese adults with DM [28].

To our knowledge, only one study in Hong Kong assessed the DQI-I relationship with NAFLD and indicated that a 10-unit decrease in DQI-I was associated with a 26% increase in the likelihood of having NAFLD [27]. Regarding the lack of studies investigating the DQI-I and DQI-R relationship with NAFLD and the discrepancy in previous findings of DQI with cardiometabolic factors, we aimed to investigate the DQI-I and DQI-R relationship with NAFLD using a case-control study among Iranian adults.

## Materials and methods

### Study population

The present study was conducted in the Metabolic Liver Disease Research Center a referral center affiliated with Isfahan University of Medical Sciences. NAFLD or non-NAFLD discrimination was ascertained using the findings of liver ultrasonography scan (USG) and gastroenterologist diagnosis. For this study, patients who were not on a special diet, did not have overt kidney or liver disease (Wilson’s disease, autoimmune liver disease, virus infection, and alcoholic fatty liver), did not have cardiovascular diseases (CVDs), severe gastrointestinal disease, malignancy, thyroid disorder, and autoimmune disease, and don’t use drugs that could be hepatotoxic or steatogenic were included. Also, participants who completed fewer than 35 FFQ items and reported under or over-reported daily energy intake (800 or 4,500 kcal/d) were excluded. Totally 225 newly diagnosed NAFLD patients and 450 controls aged 20–60 years were included for final analysis. The study details were previously reported elsewhere [6, 29].

### Dietary assessment

Dietary data collection was conducted using a previously validated and reliable semi-quantitative 168-item FFQ [30]. Trained dietitians while were blind about NAFLD status, asked participants to report their average dietary intake of each item of FFQ during the last year as follows: never or less than once a month, 3–4 times per month, once a week, 2–4 times per week, 5–6 times per week, once daily, 2–3 times per day, 4–5 times per day, and 6 or more times a day. Standard Iranian household measures were used for converting each food item’s portion size into a gram scale [31]. Different food groups are computed by summing their subgroup food items. Daily energy and nutrient intake were computed using the United States Department of Agriculture’s (USDA) Food Composition Table (FCT). Moreover, Iranian FCT was used for some local foods that were not listed in USDA FCT [32].

### Assessment of other variables

Participants’ weight and height were recorded by standard protocols. BMI was calculated as weight (kg) divided by height in square meters (*m*^2^). Information about age, sex, education level, family size, house acquisition, foreign travel, and, and smoking status was gathered via a demographic questionnaire.

The combination of five variables, including education (academic = 1 and non-academic education = 0), family size (≤ 4 people = 1, > 4 people = 0), house acquisition (house ownership = 1, Lack of ownership = 0), foreign travel (yes = 1, no = 0), and income (high = 1, low and moderate = 0) were used to compute socioeconomic status (SES) score. Based on the frequency of SES scores in our study population, participants with SES score of 0 and 1, 2, 3–5 were classified as high, moderate, and low SES, respectively.

Physical activity levels were recorded through face-to-face interviews using the International Physical Activity Questionnaire (IPAQ). All measurements were expressed as Metabolic Equivalents per week (METs/week).

### Calculation of DQI-I

The DQI-I was calculated according to the method developed by Kim et al. [14]. The score ranges from 0 to 100 and a higher score represents better diet quality. DQI-I was described using four major aspects of the diet including variety (0–20 score), adequacy (0–40 score), moderation (0–30 score), and overall balance (0–10 score). Details of the DQI-I calculation have been described elsewhere [14].

Variety score calculated from overall food group variety (meat/poultry/fish/eggs; dairy/beans; grain; fruit; vegetable) with a score of 0–15 and within-group variety for protein source (meat, poultry, fish, dairy, beans, eggs) with a score of 0–5. The adequacy score consists of 8 components with 0–5 points in each of them including vegetable, fruit, grain, fiber, protein, iron, calcium, and vitamin C. Moderation score was calculated based on total fat, saturated fat, cholesterol, sodium, and free-calorie foods that each item scored 0–6 points based on their described cut-points. The overall balance was created by macronutrient ratio (carbohydrate:protein: fat) and fatty acid ratio (PUFA:MUFA: SFA) which scored by 0–6 and 0–4 points, respectively.

### Calculation of DQI-R

The DQI-R was computed based on the method described by Haines et al. [12]. They used ten components with 0–10 score for each of them including dietary intake of total fat, saturated fat, cholesterol, fruit, vegetables, grains, calcium, iron, dietary diversity, and dietary moderation.

Dietary diversity computed considering four main food groups with the maximum score of 2.5 including grains (7 subgroups), vegetables (7 subgroups), fruits (3 subgroups), and meat and dairy (7 subgroups) based on the cut-point equal to ¼ serving daily intake for each subgroup. We calculated the moderation score using three components with a maximum score of 2.5 for each of them including added sugar, discretionary fat, and sodium intake. As we did not have information on alcohol consumption to consider under the aspect of ‘moderation’. Therefore, the range of moderation score was 0 to 7.5 instead of 0 to 10 as originally proposed in the calculation. Totally DQI-R score ranged from 0 to 97.5 instead of 0 to 100. Details of the DQI-R calculation have been described elsewhere [12].

### Statistical analysis

All statistical analysis was performed using Statistical Package Software for Social Science, version 21 (SPSS Inc., Chicago, IL, USA). The normality of variables was assessed by the Kolmogorov-Smirnov test and histogram chart. Population characteristics and dietary data were expressed as mean ± SD or median (25–75 interquartile range) for quantitative variables and percentages for qualitative variables. Comparing differences between cases and controls for continuous and categorical variables were assessed using independent sample t-test and chi-square test, respectively.

Participants were classified into tertiles based on the DQI-I, DQI-R, and some of their component cut-points among the control group. Also, the frequency of SD of mean intake of DQI-I, DQI-R, and some of their components was computed for each study participant. Multivariable logistic regression was performed to investigate the relationship between DQI-I, DQI-R, and some of their components and the odds of NAFLD. Analyses were conducted in a crude model, age and sex-adjusted model, and finally adjusted for BMI, physical activity, smoking, SES, and dietary intake of energy. The odds ratio (OR) and 95% confidence interval (CI) of NAFLD across tertiles of DQI-I, DQI-R, and some of their components were reported. We determined one SD of the DQI-I and DQI-R for individuals using SPSS software, which were considered as new variables named ZDQI-I and ZDQI-R in the data set. Then, the association of increasing each SD of DQI-I and DQI-R with the odds of NAFLD was determined using logistic regression with the adjustment of the effect of potential confounders. *P*-values < 0.05 were considered statistically significant.

## Results

The mean ± SD of DQI-I and DQI-R in all study populations were 66.6 ± 8.3 (66.8 ± 7.9 in cases and 66.4 ± 8.5 among controls) and 76.2 ± 13.3 (75.0 ± 13.0 in cases and 76.8 ± 13.4 among controls), respectively.

Table 1 shows the general information and scores of DQI-I and its components (per 1000 kcal of energy intake) among cases and controls. Compared to the control group, NAFLD patients had significantly higher BMI, % smoking, family size, foreign travel, income, and better SES (*P* < 0.01), whereas had lower physical activity and house ownership (*P* < 0.01). For DQI-I and its components, Compared to the control group, NAFLD patients had a lower total variety score, a lower score of overall food group variety, and a higher score of variety for protein source (*P* < 0.01). Also, in NAFLD patients, total adequacy score, and adequacy scores for vegetables, fruits, fiber, protein, iron, calcium, and vitamin C were lower than the control group (*P* < 0.01). Furthermore, the total moderation score and moderation scores for cholesterol and free-calorie foods, and the total score of DQI-I in the case group were lower than the controls (*P* < 0.05).

**Table 1:** General information and scores of diet quality index-international (DQI-I) and its components among cases and controls

Variables	Cases (n = 225)	Controls(n = 450)	***P***-value*
**Demographic data**			
Age, (year)	38.3 ± 8.7	37.8 ± 8.9	0.296
Male, (%)	55.6	51.8	0.354
Body mass index (Kg/m^2^)	30.5 ± 4.0	25.0 ± 3.0	< 0.001
Physical activity (MET/min/week)	1119 ± 616	1590 ± 949	< 0.001
Smoking (yes, %)	7.1	2.7	0.006
Education level (Bachelor and higher, %)	44.9	47.8	0.478
Family size (> 4 member, %,)	85.5	57.3	< 0.001
House acquisition (yes, %)	61.8	71.8	0.008
Foreign travel (yes, %)	23.6	12.0	< 0.001
Income (high, %)	24.9	4.7	< 0.001
Socio economic status (%)			< 0.001
Low (%)	21.3	31.6	
Middle (%)	33.8	40.4	
High (%)	44.9	28.0	
**DQI-I components (per 1000 Kcal)**			
***Variety score***	7.51 ± 1.80	7.78 ± 2.00	0.105
Overall food group variety score	6.38 ± 1.62	6.80 ± 1.80	0.004
Variety for protein source score	1.13 ± 0.64	0.98 ± 0.68	0.007
***Adequacy score***	16.5 ± 3.7	17.8 ± 4.2	< 0.001
Vegetable group score	1.67 ± 0.64	1.98 ± 0.79	< 0.001
Fruit group score	1.86 ± 0.70	1.99 ± 0.72	0.019
Grain group score	2.11 ± 0.57	2.20 ± 0.66	0.087
Fiber score	2.21 ± 0.54	2.36 ± 0.64	0.002
Protein score	2.30 ± 0.65	2.50 ± 0.78	0.002
Iron score	2.23 ± 0.57	2.38 ± 0.70	0.006
Calcium score	2.08 ± 0.50	2.18 ± 0.52	0.014
Vitamin C score	2.04 ± 0.61	2.19 ± 0.63	0.004
***Moderation score***	5.7 ± 3.4	6.4 ± 3.8	0.020
Saturated fat score	0.96 (0.00–1.54)	0.00 (0.00–1.49)	0.517
Cholesterol score	2.51 ± 1.13	2.70 ± 1.29	0.044
Sodium score	0.00 (0.00–1.63)	0.00 (0.00–1.94)	0.093
free-calorie foods score	0.00 (0.00–1.15)	1.02 (0.00–1.44)	< 0.001
***Overall balance score***	0.81 (0.00–1.60)	0.66 (0.00–1.60)	0.699
Macronutrient ratio (carbohydrate:protein: fat) score	0.00 (0.00–1.23)	0.00 (0.00–1.18)	0.751
Fatty acid ratio (PUFA:MUFA: SFA) score	0.00 (0.00–0.69)	0.00 (0.00–0.00)	0.140
**DQI-I score** (per 1000 Kcal)	30.7 ± 8.3	33.0 ± 9.4	0.002

Data are reported mean ± SD or median (25–75 interquartile range) for continuous variables and percentages for categorical variables

*P-value was determined using the independent two-sample t-test and Chi-square test for continuous and categorical variables, respectively

Dietary data and scores of DQI-R and its components (per 1000Kcal of energy intake) in the cases and the controls are presented in Table 2. Compared to the control group, NAFLD patients had significantly lower scores of dietary cholesterol, vegetable, and iron, lower diversity of vegetables and fruits, and lower total moderation scores, lower moderation scores of added sugar and discretionary fat, and lower total score of DQI-R (*P* < 0.05), whereas energy intake in case group was significantly higher than controls (*P* < 0.01).

**Table 2:** Dietary data and scores of diet quality index-revised (DQI-R) and its components among cases and controls

Variables	Cases (n = 225)	Controls(n = 450)	***P***-value*
**Dietary intake**			
Energy intake (Kcal/d)	2315 ± 606	2170 ± 625	0.004
Carbohydrate (% of energy)	55.9 ± 7.4	55.9 ± 6.5	0.913
Protein (% of energy)	13.2 ± 2.4	13.2 ± 2.2	0.927
**DQI-R components (per 1000 Kcal)**			
Total fat score	3.23 ± 1.88	3.51 ± 2.00	0.079
Saturated fatty acids score	3.14 ± 2.08	3.23 ± 2.30	0.585
Dietary cholesterol score	4.18 ± 1.89	4.51 ± 2.15	0.044
Fruit intake score	3.14 ± 1.90	3.41 ± 1.92	0.092
Vegetable intake score	2.43 ± 1.75	3.16 ± 1.95	< 0.001
Grain intake score	3.72 ± 1.27	3.73 ± 1.48	0.973
Calcium intake score	3.50 ± 1.24	3.67 ± 1.35	0.101
Iron intake score	4.26 ± 1.27	4.52 ± 1.60	0.032
***Dietary diversity score***	3.17 ± 0.72	3.25 ± 0.79	0.185
Grains score	0.56 ± 0.18	0.58 ± 0.20	0.235
Vegetable score	0.61 ± 0.22	0.65 ± 0.23	0.018
Fruit score	0.73 ± 0.29	0.79 ± 0.32	0.032
Meat and dairy score	0.85 ± 0.22	0.85 ± 0.26	0.945
***Dietary moderation score***	2.66 ± 1.15	3.32 ± 1.33	< 0.001
Added sugar score	1.10 ± 0.39	1.24 ± 0.40	< 0.001
Discretionary fat score	0.65 ± 0.44	1.12 ± 0.48	< 0.001
Sodium score	0.91 ± 0.55	0.95 ± 0.63	0.386
**DQI-R score** (per 1000 Kcal)	34.1 ± 9.2	37.7 ± 10.5	< 0.001

Data are presented as mean ± standard deviation

*P-value was determined using the independent two-sample t-test

The association of DQI-I and its main components with the odds of NAFLD is indicated in Table 3. Although participants in the highest versus lowest tertiles of DQI-I, variety score, and moderation score showed significant inverse associations with the odds of NAFLD in crude and age and sex-adjusted models, after adjusting for BMI, smoking, physical activity, SES, and energy intake in the final adjusted model, there was no significant association between higher DQI-I (OR = 0.69, 95%CI(0.28–1.69); P-trend: 0.509), variety score (OR = 0.50, 95%CI(0.23–1.11); P-trend: 0.077), and moderation score (OR = 0.79, 95%CI(0.39–1.60); P-trend: 0.533) with the odds of NAFLD. However, participants in the highest vs. lowest tertile of adequacy score were associated with lower odds of NAFLD based on the crude model (OR = 0.43, 95%CI(0.28–0.65); P-trend < 0.001), age and sex-adjusted model (OR = 0.42, 95%CI(0.28–0.64); P-trend < 0.001), and the final adjusted model (OR = 0.16, 95%CI(0.06–0.40); P-trend < 0.001). According to results reported for three models of logistic regression analyses, the score of overall balance was not related to the odds of NAFLD.

**Table 3:** The OR (95%CI) of NAFLD across tertiles of DQI-I and its main components and also per one SD of scores in 1000 Kcal of energy intake among the study population

	OR (95%CI) of NAFLD			*P* for trend	Per one SD	***P***-value
	T1	T2	T3			
**DQI-I**						
Median score, SD	24.20	31.38	41.53	-	9.15	-
Case/Control	98 / 150	65 / 150	62 / 150	-	225 / 450	-
Crude model	1.00 (Ref)	0.66 (0.45–0.97)	0.63 (0.42–0.93)	0.024	0.77 (0.65–0.91)	0.003
Model 1*	1.00 (Ref)	0.66 (0.44–0.97)	0.62 (0.42–0.92)	0.021	0.76 (0.64–0.91)	0.002
Model 2^†^	1.00 (Ref)	0.69 (0.34–1.36)	0.69 (0.28–1.69)	0.509	0.63 (0.40–0.98)	0.040
**Variety**						
Median score, SD	6.02	7.60	9.61	-	1.99	-
Case/Control	89 / 150	77 / 149	59 / 151	-	225 / 450	-
Crude model	1.00 (Ref)	0.87 (0.59–1.27)	0.65 (0.44–0.98)	0.040	0.87 (0.74–1.03)	0.117
Model 1*	1.00 (Ref)	0.86 (0.59–1.26)	0.65 (0.43–0.97)	0.035	0.87 (0.74–1.03)	0.107
Model 2^†^	1.00 (Ref)	0.91 (0.49–1.70)	0.50 (0.23–1.11)	0.077	0.93 (0.67–1.28)	0.673
**Adequacy**						
Median score, SD	13.77	17.48	21.93	-	4.10	-
Case/Control	111 / 150	66 / 150	48 / 150	-	225 / 450	-
Crude model	1.00 (Ref)	0.59 (0.40–0.86)	0.43 (0.28–0.65)	< 0.001	0.71 (0.60–0.85)	< 0.001
Model 1*	1.00 (Ref)	0.58 (0.40–0.86)	0.42 (0.28–0.64)	< 0.001	0.71 (0.60–0.84)	< 0.001
Model 2^†^	1.00 (Ref)	0.44 (0.22–0.88)	0.16 (0.06–0.40)	< 0.001	0.39 (0.24–0.65)	< 0.001
**Moderation**						
Median score, SD	2.89	5.66	9.46	-	3.75	-
Case/Control	91 / 150	74 / 148	60 / 152	-	225 / 450	-
Crude model	1.00 (Ref)	0.82 (0.56–1.20)	0.65 (0.43–0.96)	0.034	0.81 (0.68–0.96)	0.020
Model 1*	1.00 (Ref)	0.81 (0.55–1.19)	0.63 (0.42–0.95)	0.027	0.80 (0.68–0.95)	0.015
Model 2^†^	1.00 (Ref)	1.13 (0.64–1.99)	0.79 (0.39–1.60)	0.533	0.83 (0.61–1.13)	0.260
**Overall balance**						
Median score, SD	0.00	0.85	2.04	-	1.11	-
Case/Control	80 / 212	68 / 88	77 / 150	-	225 / 450	-
Crude model	1.00 (Ref)	2.04 (1.36–3.07)	1.36 (0.93–1.98)	0.134	1.03 (0.88–1.21)	0.698
Model 1*	1.00 (Ref)	2.00 (1.33–3.02)	1.32 (0.89–1.93)	0.198	1.01 (0.86–1.19)	0.865
Model 2^†^	1.00 (Ref)	2.24 (1.22–4.10)	1.64 (0.95–2.82)	0.070	1.14 (0.90–1.43)	0.265

^*^ Model 1: adjusted for age and sex

^†^ Model 2: adjusted for model 1 and body mass index, smoking, physical activity, socio-economic status, and dietary intake of energy

In Table 3, the OR of NAFLD per each SD increment of DQI-I and its main components in different adjusted models are presented. Each SD increment of DQI-I and adequacy score was related to lower odds of NAFLD in all adjusted models; in the fully adjusted model, the OR (95%CI) of NAFLD per one SD increment of DQI-I and adequacy score were 0.63 (0.40–0.98) and 0.39 (0.24–0.65), respectively (*P* < 0.05). In the crude and age and sex-adjusted models, per one SD increment of moderation score, the odds of NAFLD significantly decreased, however, in the final adjusted model the significant association disappeared (OR = 0.83, 95%CI: 0.61–1.13; *P*-value: 0.260). In three models of logistic regression analysis, no significant association was observed between one SD increment of variety and overall balance scores and odds of NAFLD; in the multivariable model, the OR (95%CI) of NAFLD per one SD increment of variety and overall balance scores were 0.93 (0.67–1.28) and 1.14 (0.90–1.43), respectively (*P* > 0.05).

Table 4 shows the OR (95%CI) of NAFLD across tertiles of DQI-R and its main components and also per one SD of scores in 1000 Kcal of energy intake. Higher scores of DQI-R and dietary moderation score were associated with lower odds of NAFLD in all regression models; The OR (95% CI) of NAFLD for participants in the highest vs. lowest tertiles of DQI-R in the crude and final models respectively were 0.39 (0.25–0.59), P-trend:<0.001 and 0.24 (0.10–0.53), P-trend:<0.001. Also The OR (95% CI) of NAFLD for participants in the highest vs. lowest tertiles of dietary moderation score in the crude and final adjusted models were 0.34 (0.22–0.52), P-trend < 0.001 and 0.17 (0.07–0.43), P-trend < 0.001, respectively. However, in each three models of logistic regression analysis, the higher score of dietary diversity was not associated with the odds of NAFLD; in the final adjusted model, the OR (95%CI) of NAFLD in the highest vs. lowest tertile of dietary diversity score was 0.99 (0.46–2.15), P-trend: 0.920.

**Table 4:** The OR (95%CI) of NAFLD across tertiles of DQI-R and its main components and also per one SD of scores in 1000 Kcal of energy intake among the study population

	OR (95%CI) of NAFLD			***P*** for trend	Per one SD	***P***-value
	T1	T2	T3			
**DQI-R**						
Median score, SD	27.14	36.98	47.48	-	10.28	-
Case/Control	110 / 150	72 / 150	43 / 150	-	225 / 450	-
Crude model	1.00 (Ref)	0.65 (0.45–0.95)	0.39 (0.25–0.59)	< 0.001	0.68 (0.57–0.81)	< 0.001
Model 1*	1.00 (Ref)	0.64 (0.44–0.93)	0.37 (0.24–0.57)	< 0.001	0.67 (0.56–0.80)	< 0.001
Model 2^†^	1.00 (Ref)	0.42 (0.22–0.79)	0.24 (0.10–0.53)	< 0.001	0.49 (0.34–0.70)	< 0.001
**Dietary diversity**						
Median score, SD	2.46	3.16	3.97	-	0.77	-
Case/Control	76 / 147	86 / 152	63 / 151	-	225 / 450	-
Crude model	1.00 (Ref)	1.09 (0.74–1.60)	0.80 (0.53–1.20)	0.288	0.89 (0.76–1.05)	0.198
Model 1*	1.00 (Ref)	1.09 (0.74–1.61)	0.80 (0.53–1.21)	0.302	0.89 (0.76–1.05)	0.201
Model 2^†^	1.00 (Ref)	1.38 (0.75–2.54)	0.99 (0.46–2.15)	0.920	0.86 (0.64–1.16)	0.341
**Dietary moderation**						
Median score, SD	2.04	3.12	4.45	-	1.31	-
Case/Control	121 / 149	62 / 151	42 / 150	-	225 / 450	-
Crude model	1.00 (Ref)	0.50 (0.34–0.74)	0.34 (0.22–0.52)	< 0.001	0.55 (0.46–0.67)	< 0.001
Model 1*	1.00 (Ref)	0.50 (0.34–0.73)	0.34 (0.22–0.52)	< 0.001	0.55 (0.45–0.67)	< 0.001
Model 2^†^	1.00 (Ref)	0.42 (0.22–0.80)	0.17 (0.07–0.43)	< 0.001	0.33 (0.21–0.51)	< 0.001

^*^ Model 1: adjusted for age and sex

^†^ Model 2: adjusted for model 1 and body mass index, smoking, physical activity, socio-economic status, and dietary intake of energy

Furthermore, results of Table 4 showed that each SD increment of DQI-R and dietary moderation scores were associated with lower odds of NAFLD in all adjusted models; in the final adjusted model, the OR (95%CI) of NAFLD for DQI-R and dietary moderation score were 0.49 (0.34–0.70), *P*-value:<0.001 and 0.33 (0.21–0.51), *P*-value:<0.001, respectively. However, one SD increment in dietary diversity score was not associated with NAFLD in any of the logistic models; in the final adjusted model, the OR (95%CI) of NAFLD per one SD of dietary diversity score was 0.86 (0.64–1.16), P-value: 0.341.

## Discussion

The current case-control study suggested that a diet with higher scores of DQI-I and DQI-R was associated with lower odds of NAFLD among Iranian adults. Also, the higher score of adequacy (as a DQI-I component) and higher score of dietary moderation (as a component of DQI-R) were related to lower odds of NAFLD. However, we observed no significant association between scores of variety, moderation, and overall balance as components of DQI-I and dietary diversity as a component of DQI-R with odds of NAFLD.

Our findings are consistent with the results of one observational study that assessed the possible relationship between DQI-I and the risk of NAFLD; the Chan et al. study reported that higher DQI-I was associated with a reduced likelihood of NAFLD in Hong Kong adults [27]. Also, the results of the current study are somewhat comparable with the results of investigations that focused on the association of DQI and risk of cardiometabolic disorders; consistent with our findings, the Setayeshgar et al. study indicated that a 10-unit improvement in DQI-I score was associated with lower gain in central fat mass and body fat percent after a 2-year follow-up in Canadian children. Also, they showed that among DQI-I components including variety, adequacy, moderation, and overall balance, only a higher adequacy score was associated with lower gain in fat mass index and body fat percent [25]; such a finding was also observed in our study and among DQI-I components, only higher adequacy score was related with lower odds of NAFLD. In addition, Funtikova et al. study suggested that higher DQI related to a higher level of high-density lipoprotein cholesterol (HDL-C), whereas an inverse association was observed between DQI and levels of waist circumference (WC), triglycerides (TG), and the TG to HDL-C ratio during a 10-year follow-up in adults [21]. The above-mentioned studies showed that higher DQI-I is a good instrument for predicting the reduction of body fat accumulation and hypertriglyceridemia. However, it seems that DQI-I has not been able to adequately estimate the risk of obesity and high BMI, because, in five previous studies among Iranian, Chinese, Canadian, and Spanish populations, no significant association was found between the higher score of DQI-I and BMI and obesity [21, 25, 28, 33].

The results of the present study suggested that the moderation score of DQI-I can be a good indicator of detrimental nutrients and foods that may be related to NAFLD risk. Despite the inverse association of this score with odds of NAFLD in the age and sex-adjusted model, it seems that its effects were not as strong as the need to overcome the BMI and other confounders’ effects. However, an observational study conducted on Iranian patients with a history of myocardial infarction suggested that a higher moderation score of DQI-I was associated with a better lipid profile and lower WC [34]. In general, although the available limited results support the moderation score of DQI-I as a protective factor to prevent metabolic disorders and diseases, the results of our study suggest that the conclusion about the positive effect of this DQI-I component on reducing the NAFLD risk is still premature and there is a need to do more research on this subject.

In the current study, the variety score of DQI-I was not different among cases and controls, however, its components paradoxically were different in cases and controls, and by the way that NAFLD patients consumed more diverse protein sources, whereas consumed lower diverse food groups. This issue leads to a decrease in the strength of this index in observing a significant relationship with NAFLD in the final adjusted model of covariates. Controversy on the possible effect of the variety score of DQI-I on metabolic factors was observed in the Sharifi et al. study, as the score of DQI-I was not linked to levels of BMI and WC, whereas a higher score of this dietary index had a positive association with HDL-C and TGs and a negative association with LDL-C [34]. Also, generally different dietary diversity scores were assessed in two previous systematic reviews and meta-analyses and their pooled results showed no significant association between dietary diversity score and BMI, obesity, and lipid profile except for TGs [35, 36].

In the current study, the overall balance score of DQI-I and its components including the macronutrient ratio and the fatty acid ratio was not different between the case and control groups, and higher overall balance showed no significant association with odds of NAFLD. Definitely, total calorie intake has the main role in body weight regulation and consequently body composition [37]. However, the dietary macronutrient and the fatty acid ratio have been proposed as effective factors in body composition. A previous review study showed that the effects of dietary composition on fat storage in the liver are inconsistent [38]. Also, a meta-analysis of trials reported no differences in weight loss and changes in cardiovascular and diabetes risk factors with two different dietary compositions (low CHO diets vs. isocaloric balanced diets) which is related to overall balance score [39]. Moreover, the optimal proportions of dietary macronutrients in the prevention or treatment of NAFLD patients are unknown, and maintaining a eucaloric diet along with avoiding saturated fatty acids and simple sugars is supported by the literature [37].

To the best of our knowledge, this is the first study that assessed the DQI-R and its diversity and moderation components with the odds of NAFLD and found an inverse association between DQI-R and the moderation score of DQI-R with NAFLD. Our results partly are in line with the study by Ritter et al. who showed that higher adherence to a DQI adapted for Brazilian adolescents was associated with a better cardiometabolic profile in girls with normal weight; not in overweight/obese girls. In boys with overweight/obesity, higher DQI was associated with lower concentrations of total cholesterol and low-density lipoprotein cholesterol (LDL-C), but only LDL-C remained significant in boys with normal weight [24]. Although some other studies assessed diet quality index with other chronic diseases (CDs) such as breast cancer [22], cardiovascular risk factors [40], and mortality [26], their calculated DQI had considerable differences with DQI-R developed by Haines et al. [12], which we used in our study. The moderation score of the DQI-R in our study was calculated from added sugar, discretionary fat, and sodium intake. NAFLD patients had a significantly lower score of total moderation score, added sugar, and discretionary fat than the control group. Previous studies showed the adverse effects of added sugar [41] and high fat intake [42] on NAFLD. However, in the present study dietary diversity score of DQI-R did not differ among the case and control and showed no association with NAFLD. As we mentioned above, this result is a confirmation of two previous meta-analysis that observed any significant association between dietary diversity and metabolic factors [35, 36].

The inverse relationship between a diet with higher score of DQI-I and DQI-R and NAFLD risk can be explained by some potential mechanisms and reasons; DQI-I and DQI-R have been created by considering the main food groups, some essential nutrients, and detrimental components of diet; so a higher score of these indices presents a diet rich in beneficial food groups and poor in detrimental dietary factors, which based on their unique characteristics and interaction with each other in the form of a dietary pattern, show their role in predicting the risk of metabolic disorders such as NAFLD. Higher diet quality has a generally positive correlation with all food groups and consequently more consumption of all essential and non-essential nutrients particularly fiber, protein, calcium, iron, vitamin C, w-3 fatty acids, and other antioxidant components such as vitamin A, polyphenols, and phytochemicals which previously showed an inverse association with NAFLD based on the anti-inflammatory properties, anti-oxidant effects, and prevention of adiposity [5–8, 29, 43]. On the other hand, it is negatively correlated with total fat, SFA, cholesterol, sodium, added sugars, and pro-inflammatory agents that showed adverse effects on metabolic factors [9, 41, 42].

The present study has some strengths; this is the first study that reported an association between DQI-R and its components and the odds of NAFLD in Iranian adults. Another strength is that dietary intake was assessed using locally validated questionnaires among the Iranian population, which were administered by trained and experienced dietitians through face-to-face interviews which minimized measurement errors. We acknowledge some limitations in the present study. First, because of the case-control design of this study, it is not possible to suggest a causal relationship. Second, the inevitable measurement error of FFQ or other unknown confounders may also affect our results. Third, another limitation of the current study was that the cases and controls were not matched based on important variables, such as age, gender, and BMI, however, it should be noted based on results reported in Table 1, no significant difference was observed in age and sex between the cases and controls. Also, the potential confounding effect of these variables mentioned above along with some other variables was controlled in the multivariable regression analysis. Finally, we did not have data on menopausal status, the number of pregnancies, hormonal conditions of participants, genetic data, etc. for further investigations.

## Conclusions

In summary, the findings of this case-control study revealed a possible protective role of a diet with a higher score of DQI-I and DQI-R against the odds of NAFLD. Future studies using larger sample sizes and prospective designs need to address the DQI-I and DQI-R and their components with NAFLD and other chronic diseases.

## Data Availability

The datasets analyzed in the current study are available from the corresponding author on reasonable request.

## Abbreviations

BMI: body mass index
CDs: chronic diseases
CI: confidence interval
CVDs: cardiovascular diseases
DALYs: disability-adjusted life years
DM: diabetes
DQI: diet quality index
DQI-I: DQI International
Fats: fatty acids
FCT: Food Composition Table
FFQ: food frequency questionnaire
HDL-C: high-density lipoprotein cholesterol
IPAQ: International Physical Activity Questionnaire
LDL-c: low-density lipoprotein cholesterol
METs: Metabolic Equivalents
MUFA: mono-unsaturated saturated fatty acid
NAFLD: non-alcoholic fatty liver diseases
OR: odds ratio
PUFA: polyunsaturated saturated fatty acid
SD: standard deviation
SES: socioeconomic status
SFA: saturated fatty acid
USDA: United States Department of Agriculture’s
USG: liver ultrasonography scan
WC: waist circumference
